# Progress in Surgery

**Published:** 1898-02-26

**Authors:** 


### Progress in Surgery.

### GENITO-URINARY SURGERY.

(*Continued from p.* 364.)

**Incontinence of Urine.**—Nocturnal incontinence is most successfully treated by avoiding fluids at supper-time, and administering tincture of rhus aromatica, or by cold douching of the back just before going to bed.[1] Paralytic incontinence in the female has been successfully treated by diverting the urethra into the rectum within the sphincter; and in one case, when both urethra and rectum were paralysed, Wm. Alexander relieved the patient by performing suprapubic cystostomy and closing the natural urethra. A simple apparatus is then sufficient.[2]

**Cystitis.**—A review of recent work on the bacteriology of cystitis occurs in the *Practitioner* for July, 1897. At least two distinct types of cystitis are recognisable, associated with acid and alkaline urine respectively. In the acid variety there is a great tendency for the inflammation to spread to the kidneys, causing suppuration there. It is a serious condition, and is due to the bacillus coli communis. In the alkaline type some organism, capable of decomposing urea, must be present—*e.g.*, the diplococcus ureæ liquefaciens, the proteus Hanser, the bacillus pyocyaneus, &c. Acid cystitis occurs usually in girls, as the female urethra is more readily infected by the bacillus coli communis.

Ramond has treated tuberculous cystitis by injecting sterilised air into the bladder, on the same lines as the treatment of tuberculous peritonitis by abdominal incision and exposure to air.

Professor Nicolaier speaks highly of the internal administration of urotropin, a compound of formic aldehyde with ammonia.

Reginald Harrison points out the widely-spread infection in gonorrhœa, as shown by the invasion of the bladder and reinfection of the urethra, and recommends free irrigation of the bladder with Condy's fluid.[3]

**Vesical Calculus.**—Lithotrity still holds its own as perhaps the best operation for stone in the bladder. Herbert Milton (Cairo) has operated on 550 cases, and during the last four years, with greater experience, has practically abandoned suprapubic lithotomy in favour of lithotrity for large stones, and has successfully removed a calculus by this means weighing 16 ounces. He prefers Bigelow's operation to perineal lithotrity, as his results are better, with a lower mortality and fewer after-complications.[4] Most surgeons, with less experience, would prefer the cutting operation for large stones; and suprapubic cystotomy is indicated in children below the age of puberty, also in old men with enlarged prostates. When the suprapubic operation is indicated there are few cases in which primary union of the bladder can be secured. Sterlin lays stress on the advantages of getting the bladder aseptic if possible, and using irrigations twice a day. For this purpose a drainage-tube is passed into the bladder, and connected to a soft catheter passed per urethram. Drainage and irrigation are thus possible both ways.[5] Bennett is of opinion that, under ordinary circumstances, a certain cure is more likely to be effected by suprapubic lithotomy than by any other method[6]; and Stanmore Bishop holds that the operation should be done aseptically, and union by first intention aimed at.[7] Dr. Frederick Barker still does lateral lithotomy for children, and claims to have done over a hundred consecutive cases without a death.[8]

**Vesical Tumours.**—A carcinoma of the bladder, together with part of the bladder-wall, was removed by Dr. Kammerer with immediate suture of the bladder. The sinus had quite healed after eight months, and no recurrence could be detected. Dr. Willie Meyer reported a similar case.[9] Nitze, of Berlin, has described a new form of cystoscope for the employment of a snare in the bladder cavity under full light.[10] Küster records two successful cases of partial extirpation of the bladder with tumours involving the ureter.[11]

**Extroversion of the Bladder.**—Several new operations have lately been devised for this condition. The method of Mikulicz comprises three stages: (*a*) Formation of the bladder by lateral incisions at the edges of the mucuous membrane, the edges of which are united together in the middle line; (*b*) formation of urethra and penis by dissecting up lateral flaps, and uniting them over a catheter; and (*c*) closure of the neck of the bladder by the union of the foregoing.[12] Mr. Harrison, at the Medical Society of London, in April, 1897, recorded a case which he had treated in a novel way. He first of all removed one of the kidneys, and after an interval long enough for the other kidney to hypertrophy the remaining functional ureter was transplanted into the loin. The urine then flowed with much less mucus, and the vesical extroversion cicatrised. Mr. R. W. Murray (Liverpool) recommends an operation which, by avoiding the inversion of skin-covered flaps, prevents the formation of phosphatic deposits about the hairs. Two lateral flaps are dissected up and raised, so that when united in the middle line the bladder is bridged over by them. The flaps are united in the middle line in a condition of eversion by double sets of sutures.[13]

**Vesico-Vaginal Fistulæ.**— Dr. Ricard lays stress on the value of freshening the edges by splitting, and he places sutures only in the vaginal portion.[13] The opening may be first closed like a purse by deep sutures, and then superficial sutures are used at the vaginal aspect.

**Scrotum, &c.**—Halban reports a hæmatocele which suppurated spontaneously owing to the presence of the pneumococcus.[14] Dr. James Miller (Lambeth) speaks much in favour of injection of hydroceles with perchloride of mercury, which he has found very effectual.[15]

Dr. Pilate employs free irrigation of the tunica vaginalis, with large quantities of a 3 per cent. solution of carbolic acid after simple puncture.[16] It is particularly successful in the cases of hydrocele in young people.

Several cases of elephantiasis of the scrotum and filarial hydrocele have been published. Professor Le Dentu has recognised a condition of the testicle resembling tertiary syphilis which he has proved to be of filarial origin, being associated with filarial lymphangioma of the inguinal canal and elephantiasis.[17] Havelock Charles recommends covering the bared surface after removal of the scrotal tissue in elephantiasis with flaps of skin obtained by sliding from the thighs.[18] A complete record of a very interesting case of filariasis with post-mortem appearances may be found in the *British Medical Journal*, April 24th, 1897.

[1] Practitioner, August, 1897. [2] Lancet, July 3rd, 1897. [3] N. Y. Med. Record, July, 1897. [4] N. Y. Med. Record, Sep. 18, 1897. [5] Deut. Zeitsch für Chir., XLIV., Heft 3, 4. [6] Clinical Journ., Aug. 4th, 1897. [7] Practitioner, Oct., 1897. [8] Clin. Journ., Jan. 2nd, 1897. [9] Trans. of N. Y. Surg. Soc., April 28th, 1897. [10] N. Y. Med. Record, Sept. 18th, 1897. [11] Annals of Surgery, May, 1897. [12] Annals of Surgery, Sep., 1897; B. M. J., June 12th, 1897. [13] Med. Week, Mar. 19th, 1897. [14] Ep. B. M. J., May, 1897. [15] Lancet, Sept. 4th, 1897. [16] Med. Week, Aug. 13th, 1897. [17] Med. Week, Sep. 10th, 1897. [18] Epit. B. M. J., April 24th, 1897.

